# Knowledge, attitudes, and values among physicians working with clinical genomics: a survey of medical oncologists

**DOI:** 10.1186/s12960-017-0218-z

**Published:** 2017-06-27

**Authors:** Peter Chow-White, Dung Ha, Janessa Laskin

**Affiliations:** 1 Simon Fraser University, Burnaby, Canada; 2 British Columbia Cancer Agency, Columbia, Canada

**Keywords:** Medical big data, Cancer genomics, Cancer clinical trials, Genomic literacy, Applied cancer genomics, Health education, Health professionals, Genomic technologies, Genomic sciences

## Abstract

**Background:**

It has been over a decade since the completion of the Human Genome Project (HGP), genomic sequencing technologies have yet to become parts of standard of care in Canada. This study investigates medical oncologists’ (MOs) genomic literacy and their experiences based on their participation in a cancer genomics trial in British Columbia, Canada.

**Methods:**

The authors conducted a survey of MOs from British Columbia, Canada (*n =* 31, 52.5% response rate), who are actively involved in a clinical genomics trial called Personalized Onco-Genomics (POG). The authors also measured MOs’ level of genomic knowledge and attitudes about clinical genomics in cancer medicine.

**Results:**

The findings show a low to moderate level of genomic literacy among MOs. MOs located outside the Vancouver area (the major urban center) reported less knowledge about new genetics technologies compared to those located in the major metropolitan area (26.7 vs 73.3%, *P <* 0.07, Fisher exact test). Forty-two percent of all MOs thought medical training programs do not offer enough genomic training. The majority of the respondents thought genomics will have major impact on drug discovery (67.7%), and treatment selection (58%) in the next 5 years. They also thought the major challenges are cost (61.3%), patient genomic literacy (48.3%), and clinical utility of genomics (42%).

**Conclusions:**

The data suggest a high need to increase genomic literacy among MOs and other doctors in medical school training programs and beyond, especially to physicians in regional areas who may need more educational interventions. Initiatives like POG play a critical role in the education of MOs and the integration of big data clinical genomics into cancer care.

**Electronic supplementary material:**

The online version of this article (doi:10.1186/s12960-017-0218-z) contains supplementary material, which is available to authorized users.

## Background

Physicians are increasingly working with genomic big data in their research and clinical work. Genomic sequence data helps scientists understand the molecular causes of diseases such as cancer [1, 2]. Since the completion of the Human Genome Project (HGP) in 2003, scientists promoted a genomic revolution in which genomics would create radical breakthroughs in scientific and biomedical practice [3]. At the end of the decade, scholars raised questions about the pace of progress of these lofty promises translating into clinical action [4]. Despite massive funding and research by both public and private agencies for several decades, clinical application of genomic technologies is still facing many risks and hurdles [5]. Physicians have begun to adopt genomic data and technologies into clinical practice, and single gene tests have become integrated into the standard of care for the treatment of some specific cancer types. Like many big data technologies, discovery takes time and translation from scientists to doctors is typically incremental.

There are a number of challenges to the adoption of genome sequencing into routine practice such as doctors’ attitudes toward genomics, their level of genomic literacy, and experiences working with clinical genomic trials. One of the main challenges is a lack of familiarity and understanding of cancer genomics among healthcare professionals. Physicians often report their level of genomic knowledge is inadequate in order to make treatment decisions based on a patient’s genome sequencing information [6–8]. The current status of medical genomic education in Canadian medical school is also limited [9]. Despite limited genomic knowledge, physicians express a positive attitude toward increasing their genomic knowledge, and a desire to adopt genomics into their practices [10–14]. Other studies have shown, otherwise, a mixed attitude toward genomics and their willingness to adopt the technologies [15–17].

This is an early stage of adoption as genomics moves from scientist stakeholders to medical practitioners and the public [18]. Put another way, genomics is moving from the research bench to the clinical bedside. It is critical to update our understanding of doctors’ perspectives and experiences during the adoption process. This knowledge can be fed back to clinical trial researchers to help develop clinical genomic technologies. The purpose of this study was to understand doctors’ genomic literacy and help direct pedagogy for medical students and practicing doctors.

## Methods

We conducted a web-based survey from Nov 2015 to April 2016 with a sample of Canadian physicians who are medical oncologists. The Department of Research Ethics Review Board (DORE) at Simon Fraser University approved the study (Approval #2014s0172).

### Sample

We surveyed medical oncologists (MOs) involved in an experimental clinical genomics trial in the province of British Columbia, Canada. These specific MOs were chosen for the survey because they are investigators in a precision medicine clinical trial called Personalized Onco-Genomics (POG), led by clinicians and scientists at the British Columbia Cancer Agency (BCCA) in Canada (ClinicalTrials.gov Identifier: NCT02155621). Therefore, our study sample is a relatively select group of oncologists who have experience working with clinical genomics and a higher exposure than other physicians. The cancer clinical trial enrols patients from across the province with incurable cancers that is primarily based on stages such as incurable stage II and IV advanced cancers, who have limited or no standard treatment options available (full enrolment criteria are outlined in the trials link above and in Laskin et al. study [19]). Each patient undergoes a tumor biopsy and this sample undergoes comprehensive DNA and RNA sequencing. Genome scientists perform in-depth bioinformatic analyses comparing the normal DNA to the cancer DNA and the RNA expression in order to identify variants that may be cancer “drivers” or therapeutically actionable targets [19]. Theses analyses involve a dedicated team of genome scientists and bioinformaticians and the process from the time of consent to the generation of a report takes approximately 10 weeks; details on the methodology for this complex analytic process can be found in Laskin et al. [19] Thus, POG generates medical big data from genomes and transcriptomes, and other types of biological and medical information; each case represents 1.5 terabytes of data that needs interpretation. This is big data. POG is an interdisciplinary collaboration between physicians, medical oncologists, genome scientists, pathologists, bioinformaticians, medical geneticists, and social scientists from communication, bioethicists, and health economists. The group meets weekly to discuss two to four individual patient cases. There are three parts to the analysis. First, a MO presents an overall background of the patient, their current cancer treatment, and may ask the data analysts specific questions regarding the next therapy that would be standard for this patient. Second, a pathologist presents the tumor analysis of the cancer patient. Third, a bioinformatician/genome analyst presents genomic sequencing and a genomic pathway data and identifies potential biological pathways to be considered for a therapeutic intervention. The presentations are followed by a collective discussion and assessment for potential treatment strategy. This is different than commercial panel-based profiling tests in which more simplified versions of genomic analysis can be ordered by MOs, who then receive a report of genomic data that they must interpret for themselves. The POG meetings signify the communication culture of medicine where the meaning making of genomic results takes place through the social interactions, discussions, and communication of the multidisciplinary medical stakeholders. As of this writing, POG’s enrolment is over 900 patients and includes 50 pediatric cancer cases.

We surveyed 59 MOs who enrolled patients in POG. This represents over 60% of MOs in the BCCA network (*n =* 103) and almost 48% of the MOs in the region of British Columbia (*n =* 123). The response rate was 53%. Some MOs did not participate in the survey because they only had one patient enrolled in the program. Hence, they did not have enough experience working with POG to provide any useful inputs for the survey. Complex clinical genomics is not widely adopted at this historical point, so this survey captures a high number relative to the actual amount of doctor early adopters in Western Canada.

### Survey measures

The survey instrument is a 29-item web-based questionnaire including questions about MO genomic literacy, experiences and attitudes toward genomics, and their perceived values of POG. The survey also included questions about MOs’ demographic information such as gender, years of practicing oncology, their location, and the number of cancer patients they treat per year. We organized the survey into three sections including our respondents’ knowledge of genomics, their experiences and attitudes toward the use of genomics in oncology, and their perceived values of POG.

We undertook a number of steps to construct the survey. Clinical genomics is an emerging area, so there is little research on its application at present. We aimed to be as consistent as possible with past survey research in the area and the specifics of POG [20, 21]. We conducted a systematic review of empirical research on genomic literacy and genetic education as well as literature review of research on attitudes and experiences with genomic and genetic technologies (Ha et al., under review). We adapted existing measures from UBC Physician Education [20] and Middleton et al. [21], because these two studies have similar research objectives of examining the level of knowledge, attitudes, and experiences of physicians working with clinical genomics. We also consulted with the leadership team of POG, which includes a genome scientist, a bioinformatician, and three MOs. We interviewed the five co-leads with a semi-structured and open-ended protocol to understand what they want to know from the MOs and collaborate to co-construct the survey. The goal of these semi-structured interviews with the project principals of POG is to understand their thoughts and interests in the survey. As a result, we incorporated the findings from the interviews to construct the survey questions. Although different interviewees have different perspectives on what they want to know from the POG survey, those perspectives are inter-correlated with each other. The main interests they want to find out from POG survey include four main themes: (1) the clinical values of POG in which whether POG help change their decision-making process or management plan for their cancer patient treatment; (2) the oncologists’ expectations when coming to POG and experiences after collaborating with POG; (3) the knowledge or understanding about genomics among the oncologists; and (4) the communication process of POG. Taken together, we constructed the survey protocol based on the extensive literature review, systematic review, and semi-structured interviews.

After we developed the protocol, we piloted the survey with a small sample (*n =* 9) of MOs at POG. We gave the MOs in the pilot survey an opportunity to provide feedback into improving the survey. We incorporated the feedback from our pilot survey respondents and revised the survey protocol. We then validated the survey protocol with a clinician who is an expert in clinical genomics. We relied on her expertise to build content and scientific validity of the instruments and to ensure the instruments would make sense to their communicative cultural context. Overall, this survey is a co-construction between social scientists and medical experts including a genome scientist, a bioinfomatician, and three clinicians.

### Statistical analyses

We analyzed categorical response frequencies to report our descriptive statistics. Conceptually related sets of rating scaled responses were subjected to within-subjects repeated measures analysis of variance (ANOVA) to compare the differences of their mean scores. We also performed inferential statistics between gender, location, years of practicing oncology, and number of cancer patients as independent variables with the level of genomic knowledge and POG values as dependent variables. Due to the relatively small sample, instead of using *χ* ^2^, we used Fisher exact test to examine the statistical significance of the findings.

## Results

### Participant characteristics

The results showed almost equal gender distribution between female (*n =* 15) and male (*n =* 16) MOs (Table 1). Other variables included their years of practicing oncology and their number of cancer patients per year in order to get a sense about their oncology experience. We used central tendency measurements of median for our ratio variables of “years of practicing oncology” and “number of cancer patients per year” to distribute the data equally for these two variables into two groups divided by their median. The median for “number of cancer patients per year” was 180, so we grouped the responses into two groups of less than or equal to 180 or more than 180 patients per year. Likewise, the median for “years of practicing oncology” was 12, which coincidently matched with the number of years since the Human Genome Project (HGP) was completed. We assumed that based on the impactful discoveries of the HGP, it could result in a paradigm shift in medical research and in styles of thoughts between MOs who had been practicing before and after the HGP. Location where MOs practice was also an important factor to take into account. The majority of our respondents (*n =* 13) work in Vancouver and the rest work outside Vancouver.

**Table 1:** Demographic characteristics of our population

Variables	Frequency	Percentage (%)
Gender		
Female	15	48.4
Male	16	51.6
Years of practicing oncology		
≤12 years	16	51.6
>12 years	15	48.4
Number of cancer patients in the past year		
≤180 patients	18	58.1
>180 patients	13	41.9
BCCA centres		
Abbotsford Centre	3	9.7
Fraser Valley Centre	9	29
Southern Interior	5	16.1
Vancouver Centre	13	41.9
Vancouver Island	1	3.2

### Genomic knowledge

In the first section of the survey, we explored MOs’ perceptions and level of knowledge about genomics. We asked our participants to rate their level of knowledge based on a scale of 1 = “little knowledge”, 2 = “knowledgeable”, 3 = “very knowledgeable”, and 4 = “expert” on three different topics of genomic science and technologies: (1) basic genetic principles (i.e., inherited patterns), (2) newer genetic/genomic technologies (i.e., high-throughput sequencing, genotyping and copy number variation analysis), and (3) the process of whole genome sequencing or WGS (i.e., features, eligibility criteria for sequencing, benefits, risks, and non-medical implications). The results showed the majority of the MOs ranked themselves as knowledgeable (57%) or very knowledgeable (33%) (mean = 2.36; SD = 0.66) about the topic of basic genetics principles (Table 2). Seven percent of the physicians claimed that they have little knowledge. However, the results shifted as more MOs acknowledged they had little knowledge about newer genetic technologies (50%) (mean = 1.61; SD = 0.67) and WGS process (41%) (mean = 1.77; SD = 0.76). Only one MO considered themselves to be an expert on the field of basic genetics principles and whole genome sequencing process, and no MO regarded themselves as an expert in newer genetic technologies. 45.2% of the respondents did not have enough information and knowledge to understand the POG meeting and results (Table 5). 32.3% of them did not feel confident that they could communicate POG results to their patients (Table 5). As a result, the majority of our respondents reported little or adequate knowledge about genomics (mean = 1.61–2.35; item main effect *F*(1.5,46) = 30.7, *P <* 0.0001).

**Table 2:** Physicians’ level of genomic literacy

Genomic literacy	Little knowledge (%)	Knowledgeable (%)	Very knowledgeable (%)	Expert (%)	Mean (SD)
Basic genetic principles (i.e., inherited patterns)	6.45	54.8	35.5	3.2	2.36 (0.66)
Newer genetic/genomic technologies (i.e., high-throughput sequencing, genotyping and copy number variation analysis)	48.4	41.9	9	0	1.61 (0.67)
The process of whole genome sequencing (i.e., features, eligibility criteria for sequencing, benefits, risks, and non-medical implications)	38.7	45.2	9.7	3.2	1.77 (0.76)

We also asked the respondents to rate the sufficiency level of genomic education and training in medical schools. We were aware that the majority of our participants graduated from medical schools at least 5–10 years ago. However, many of them are professors in medicine and genetics or supervising medical students at a local medical school and are familiar with current medical school curriculum. The results showed that the majority of our respondents either do not know (54.8%) or think medical training (4–5 years) program did not sufficiently (42%) prepare students with enough genomic materials or training. Likewise, the majority of the MOs also thought there was not enough genomic training during their specialized medical training (54.8%), residency or fellowship (67.8%), or postgraduate medical training (58%).

We also explored how important it is for MOs to improve their knowledge of clinical applications of genomic science and technologies by asking them to rate on a scale of 1 = “unimportant”, 2 = “somewhat important”, 3 = “important”, and 4 = “very important”. The data showed that 45% of our respondents (*n =* 14) considered it very important to improve their genomic knowledge. Another majority of our respondents (39%) only thought it was “important” to improve genomic knowledge. Even though none of MOs consider updating their genomic knowledge unimportant, 16% of the respondents (*n =* 5) considered improving genomic knowledge only somewhat important. In sum, the majority of MOs felt improving their genomic knowledge was highly important but this activity was not urgent.

Since most MOs considered it important to improve their genomic knowledge, we asked who they think should be responsible for updating them about genomics. Respondents could choose multiple answers for this question (i.e., “check all that apply”). The MOs considered themselves to bear the primary responsibility for updating genomic knowledge (84%) followed by medical training and research institutions (Additional file 1: Table A1, online only). 64.5% of the respondents felt medical schools and Genome British Columbia (an arms length provincial government funder) should hold some responsibility for education. An interesting and unexpected finding was a significant number of MOs viewed POG as a place to find effective treatment *and* learn more about genomics (Additional file 1: Table A2, online only). 93.6% of the MOs agreed that meeting with the POG team was worthwhile, as they could learn more about genomics through interpersonal channels and face-to-face exchanges with other physicians involving with clinical genomics (Table 5). Most notably, there were two respondents who collaborated with POG for the sole reason of learning more about genomic research.

We found geographic location plays a role in levels of genomic knowledge in BC. MOs who work in Vancouver reported a higher level of knowledge about genomics on average than those who work outside Vancouver. More respondents who work outside Vancouver reported little knowledge about new genetics technologies compared to those who work in Vancouver (73.3 vs. 26.7%, *P <* 0.07, Fisher exact test). Likewise, no respondents who work outside of Vancouver reported being very knowledgeable or expert in whole genome sequencing compared with those who work in Vancouver (0 vs. 30.8%, *P <* 0.09, Fisher exact test). The data showed the domain experts who reported the highest levels of knowledge about genomic technologies are located in Vancouver. Those located outside greater Vancouver, the major urban center in BC, reported lower levels of genomic knowledge on average.

### Physicians’ attitudes and experiences

We asked the respondents to envision the impact of genomic technologies on their practice in the near future. The respondents rated seven items on a scale from 1 = “no impact”, 2 = “minor impact”, 3 = “major impact”. We found 67.7% (Table 3) of the respondents envisioned that in the next 5 years genomic technologies would have major impact on drug discovery (mean = 2.68; SD = 0.48). Genomic technologies would also have major impact on helping physicians select course of treatment (58%) (mean = 2.55; SD = 0.57), and sequence whole genomes for their cancer patients (58%) (mean = 2.48; SD = 0.68). 58.1% of the respondents felt more confident making treatment decisions after becoming informed about their patients’ genome (Table 5). However, the majority of our respondents thought genomic technologies would only have a minor impact (58%) or no impact (9.7%) on making a diagnosis (mean = 2.23; SD = 0.62). 61.3% of our respondents thought genomic technologies would have a minor impact on extending and improving lives (mean = 2.19; SD = 0.6). Overall, the majority of the MOs envisioned genomic science and technologies would have some impact on their oncology practices but nothing as major or significant (mean = 2.19–2.68; item main effect *F*(6,180) = 5.1, *P <* 0.0001).

**Table 3:** Impact of genomics on oncology practices in the next 5 years

Impact	No impact (%)	Minor impact (%)	Major impact (%)	Mean (SD)
Making a diagnosis	9.7	58	32.3	2.23 (0.62)
Drug discovery	0	32.3	67.7	2.68 (0.48)
Repurposing existing drugs	0	48.4	51.6	2.52 (0.51)
Selecting course of treatment	3.2	38.7	58.1	2.55 (0.57)
Extending and improving lives	9.7	61.3	29	2.19 (0.6)
Impact of transcriptome data	6.5	54.8	38.7	2.32 (0.6)
Impact of whole genome data	9.7	32.3	58	2.48 (0.68)

We also asked the respondents about concerns they may have about expanding genomic science and technology into their practices on a scale of 1 = “unconcerned”, 2 = “somewhat unconcerned”, 3 = “somewhat concerned”, 4 = “very concerned.” The three most concerning issues our respondents have when applying genomic science and technologies into their clinical practices are cost (61.3%) (mean = 3.58; SD = 0.56), patient comprehension of genomic science and technologies (48.3%) (mean = 3.39; SD = 0.67), and clinical usefulness of genetic data (42%) (mean = 3.26; SD = 0.78; Table 4). Overall, participants were mostly just somewhat concerned about pitfalls genomic science and technologies might bring about (mean = 2.55–3.58, item main effect *F*(7,210) = 8.03, *P <* 0.0001).

**Table 4:** Concerns about genomic science and technology

Concerns	Unconcerned (%)	Somewhat unconcerned (%)	Somewhat concerned (%)	Very concerned (%)	Mean (SD)
Clinical usefulness of genetic data (specificity/sensitivity/reliability	3.2	9.7	45.2	41.9	3.26 (0.78)
Extra effort without changing treatment	0	12.9	48.4	38.7	3.26 (0.68)
Decision-making on what results to return to patients	3.2	13	54.8	29	3.1 (0.75)
Results leading to ineffective or harmful treatment	3.2	22.6	42	32.2	3.03 (0.84)
Cost	0	3.2	35.5	61.3	3.58 (0.56)
Immaturity of genomic science and technologies	3.2	19.3	42	35.5	3.1 (0.83)
Patient comprehension of genomic science and technologies	0	9.7	42	48.3	3.39 (0.67)
Unexpected germline findings	6.4	42	42	9.6	2.55 (0.77)

## Discussion

### Low level of genomic literacy

The results suggest there is a need for better strategies and guidelines for enhanced genomic education among MOs specifically and doctors more generally. Most MOs were comfortable with basic genetic principles. However, they felt much less comfortable when it came to more complex cancer genomics. Most of the respondents agreed it is important for them to improve their understanding of clinical applications of genomic sciences and technologies, which reflects other survey findings [6–17]. A number of venues could address this knowledge gap. Medical schools are the first educational point for clinical genomics. However, respondents reported that the current medical training does not sufficiently prepare future doctors with enough genomic materials and education. Arguably, as scientific and medical discoveries emerge, it may be difficult for medical schools to keep up with all new advancements. However, the results from this survey show MOs feel genomics will have a major impact on their work. As a result, medical schools should focus on teaching future doctors how to teach themselves life-long learning skills.

The results suggested MOs are looking for alternative sources to learn about genomics. MO thought that a local government funding agency, Genome British Columbia, should spend more funding on research and projects that can enhance their genomic knowledge and alleviate their genomic educational needs. Educating professionals and the public is a goal of Genome BC, so this would be a strategic opportunity to focus on. Furthermore, the data also indicated MOs collaborated with POG to find effective treatment options *and* to learn about genomic research. POG plays a pedagogical role on top of a scientific/clinical role. This suggests clinical genome projects and trials serve more than the expected discovery and application of knowledge functions. They also are places where doctors go to further their own learning. As mentioned earlier, POG is different from other models of clinical profiling in which the knowledge production of genomics takes place through interdisciplinary meetings between different medical stakeholders. These social interactions, discussions, and communication at the interdisciplinary meetings are inherently educational for clinicians to learn and improve their genomic literacy. Additionally, professional training, workshops, clinical rounds, and continuing medical education (CME)-accredited events are potential tools that can help MOs update their knowledge of genomic sciences and technologies. Furthermore, health information technology systems and other online, point-of-care tools are innovative and effective educational resources for physicians, which thereby results in better utilization of genetic information in clinical practices. For example, social media platforms such as Twitter and YouTube are low-cost and wide-reaching platforms for interactive educational tools.

### Mixed attitudes toward genomic technologies

The MOs in our sample showed a mix of attitudes toward the use of genomic technologies in clinical practices. The findings on the impact of genomics on oncology practices (Table 4) and concerns about genomic science and technology (Table 5) indicated that genomic technologies could change the way MOs understand the molecular causes of diseases by genome sequencing and personalize drugs and treatments particularly to a patient’s genome. However, the uncertainties of clinical utility and validity of genomic information are a hurdle for MOs to incorporate genomic data into their diagnosis and treatments. The reluctance to adopt genomic technologies into clinical practices could also result from the lack of genomic knowledge to analyze, evaluate, and apply genomic information. These findings were consistent with the results from Gray et al. [8] study, in which physicians, who decided not to adopt genetic testing in clinical practices or to not disclose test result, tend to have “lower genomic confidence and lower reported baseline understanding” (p 1320). As a result, the lack of genomic literacy could engender a negative attitude among physicians about the effect of genomic technologies in diagnosis and treatment and impede the adoption of genomic technologies into healthcare systems. If doctors are not on board, then it will be difficult to implement *and* develop clinical genomic technologies at the population level. On the basis of the educational deficiencies identified in this survey, the POG team has initiated applied cancer genomics symposiums for the physicians of BC to address some of these educational gaps.

**Table 5:** Physicians’ experiences and perceived values with POG

Statements	Strongly disagree (%)	Somewhat disagree (%)	Somewhat agree (%)	Strongly agree (%)	Mean (SD)
I feel more confident making treatment decisions after becoming informed about my patients’ genome	12.9	29	51.6	6.5	2.52 (0.81)
I had enough information and knowledge to understand the POG meeting and results	6.5	38.7	41.9	12.9	2.61 (0.8)
I feel confident that I could communicate POG results to my patients	9.7	22.6	61.3	6.5	2.65 (0.75)
POG added another layer of confirmation to existing indicators	12.9	29	51.6	6.5	2.52 (0.81)
Meeting with the POG team was worthwhile	3.2	3.2	61.3	32.3	3.23 (0.67)

### Geographical factors in genomic literacy

In the global era of rapid information movement on the Internet, there is a tendency to think geography is not a particularly important challenge in information gathering and learning. However, sociologists of globalization find geography matters because a central hub of innovation can attract capital, experts, and infrastructures for the development and diffusion of innovation. Consistent with this research we found geography plays an important role in variations in MOs genomic literacy. Respondents who work outside Vancouver reported lower levels of genomic knowledge than those who work in the metropolitan center. Vancouver is the central hub and a milieu of innovation for professional and educational networks connecting different medical stakeholders with medical skills and expertise. POG is an interdisciplinary group of oncologists, pathologists, bioinformaticians, bioethicists, and health economists. One of the ten principles for good interdisciplinary team work is communications strategies and structures [22]. Proximity to a metropolitan center like Vancouver, Boston, or New York can have an impact on the level of genomic knowledge possibly due to easier access to more genomic training, workshops, or conferences and other face-to-face community opportunities. As a result, it is going to be important to take into account geographic location to identify the best strategies and targets to address the educational needs. To design better genomic training pipelines, genomic scientists and policy makers should target doctors who work outside major cities and metropolitan centers. The POG team has started creating targeted educational strategies for those working in regional areas of the province. Also, they are creating “POG-casts”, which are short form educational videos for YouTube. Clinical genomics trial programs such as POG have a significant pedagogical role for working doctors and physicians to learn more about genomics through utilizing the technologies and collaborating with other medical stakeholders.

## Conclusion

The main potential limitations of this study are the raw numbers of participants and reliance on some self-report item, namely the instruments for measuring levels of genomic literacy. The sample size limited our ability to apply more inferential analyses such as logistic regression models to identify more associations between our variables. Other studies have employed tests to measure genomic literacy. We considered this option but did not pursue it because of the other goals of the survey and the limited time respondents would most likely spare to complete it. However, a strength of the study is the response rate. We surveyed 54% of all MOs in POG, which represents almost 30% of all working MOs in BC in the survey population. Fifty-four percent is a very good response rate for a survey and generalizable to the study population. Both the response rate and the sample population relative to the overall population provide a solid foundation to build on for future studies. Other strengths of the study include the rigorous, multi-step process to construct and validate the questionnaire. We consider this survey a co-production between social scientists and medical domain experts. We adapted existing items and measures from other questionnaires examining genomic knowledge of physicians [20, 21]. We also incorporated findings from the semi-structured interviews with the POG project principals to construct the survey questions. Then, the survey was assessed twice through a physician who is an expert in clinical genomics, and a pilot test of MOs with a feedback mechanism. This survey provides useful measures to assess general genomic literacy and yields interesting findings. Future research could apply our survey protocol to assess the reliability and validity of the measures and its performance compared to other measures. Another strength of our study is the consistency in our findings with other studies in the same research, which showed a low level of genomic knowledge and a mixed attitude regarding genomics [6–17]. Some might argue that physicians who work at experimental clinical trials like POG would have higher genomic knowledge than other physicians. However, majority of our respondents appeared to have low genomic literacy. This implies that other physicians outside the BCCA network are likely to have even lower levels of awareness, knowledge, and favorable attitudes toward genomic technology.

A recent report from the Secretary’s Advisory Committee on Genetics, Health, and Society (SACGHS) also pointed out a lack of basic genetic understanding among many health professionals, which in turn limited the adoption of genomic technologies into clinical practices [23]. We suggest this points to clinical genomics still in early stages of adoption where the validity and application is highly uncertain. There is a critical need to understand these early adopters, however. Technology development of any kind can be better strengthened with domain expert input at the earliest stages. If not, then the risk is creating something that does not fit the user needs or their buy in. Therefore, our findings point to a high need for substantive applied genomic education for cancer physicians specifically right now. Working doctors also need opportunities to further their education at conferences and workshops as well as self-pacing methods. Medical schools will need to address all students as genomics diffuses into different areas of health care practice. It is also important for medical schools to keep updating their curricula topics in relevance with the rapid advancement of medical genomics.

As POG is a self-funded clinical trial through BCCA, we did not examine the impact of the pharmaceutical and biotechnology industry on the clinical practices at POG. Future research could investigate the impact of the pharmaceutical industry on cancer clinical trials and any ethical issues including transparency that could be derived from this conflict of interests. Finally, our findings shed light on the current level of genomic literacy among physicians in Western Canada. Physicians who locate outside metropolitan areas tend to have lower genomic knowledge than those who work in the city. More genomic training and workshop should be offered in regional areas to physicians who need more educational interventions. Initiatives like POG play a critical role in the education of MOs and the integration of big data clinical genomics into cancer care.

## Additional file

Additional file 1: Table A1. Stakeholders responsible for updating physicians about genomics. Table A2 Goals in partnering with POG. (DOCX 12 kb)

## Abbreviations

BC: British Columbia
BCCA: British Columbia Cancer Agency
DNA: Deoxyribonucleic acid
HGP: Human Genome Project
MOs: Medical oncologists
POG: Personalized Onco-Genomics
RNA: Ribonucleic acid
SACGHS: Secretary’s Advisory Committee on Genetics, Health, and Society
SD: Standard deviation
UBC: University of British Columbia
WGS: Whole genome sequencing
