# Supplementary material for: Knowledge, attitudes, and values among physicians working with clinical genomics: a survey of medical oncologists
Source: Hum Resour Health. 2017 Jun 27;15:42. doi: 10.1186/s12960-017-0218-z (PMC5488429; doi:10.1186/s12960-017-0218-z)
Supplement: Additional file 1: Table A1. — Stakeholders responsible for updating physicians about genomics. Table A2 Goals in partnering with POG. (DOCX 12 kb) [file 12960_2017_218_MOESM1_ESM.docx]

**Additional file 1**

**Table A1.** Stakeholders responsible for updating physicians about genomics

| **Stakeholders** | ***f*** | **Percent of Cases (%)** |
| --- | --- | --- |
| Physicians should update themselves | 26 | 84 |
| Medical Schools | 20 | 64.5 |
| Genome BC | 20 | 64.5 |
| Genome Sciences Centre | 18 | 60 |
| Regional Health Authority | 8 | 26.7 |
| Ministry of Health Services | 5 | 16.7 |

**Table A2.** Goals in partnering with POG

| **Goals in partnering with POG** | ***f*** | **Percentage (%)** |
| --- | --- | --- |
| Find effective treatment | 12 | 38.7 |
| Find effective treatment  Learn more about genomics | 11 | 35.5 |
| Find effective treatment Get access to certain drug Learn more about genomic research | 6 | 19.3 |
| Learn more about genomic research | 2 | 6.5 |
